# From a drought to HIV: An analysis of the effect of droughts on transactional sex and sexually transmitted infections in Malawi

**DOI:** 10.1016/j.ssmph.2022.101221

**Published:** 2022-09-03

**Authors:** Carole Treibich, Eleanor Bell, Aurélia Lépine, Elodie Blanc

**Affiliations:** a Univ. Grenoble Alpes, CNRS, INRAE, Grenoble INP, GAEL, 38000, Grenoble, France; b Office of Health Economics, UK; c Institute for Global Health, University College London, UK; d MIT Joint Program on the Science and Policy of Global Change, Massachusetts Institute of Technology and Motu Economic and Public Policy Research, United States

**Keywords:** Malawi, HIV/AIDS, Transactional sex, Sexually transmitted infections, Climate change, Drought

## Abstract

Each year there are over 300 natural disasters globally with millions of victims that cost economic losses near USD$100 billion. In the context of climate change, an emerging literature linking extreme weather events to HIV infections suggests that efforts to control the HIV epidemic could be under threat. We used Demographic and Health Survey (DHS) data collected during the 2015–2016 harsh drought that affected several areas of Malawi to provide new evidence on the effect of an unanticipated economic shock on sexual behaviours of young women and men. We find that amongst women employed in agriculture, a six-months drought doubles their likelihood of engaging in transactional sex compared to women who were not affected by the drought and increases their likelihood of having a sexually transmitted infections (STI) by 48% in the past twelve months. Amongst men employed outside of agriculture, drought increases by 50% the likelihood of having a relationship with a woman engaged in transactional sex. These results suggest that women in agriculture experiencing economic shocks as a result of drought use transactional sex with unaffected men, i.e. men employed outside agriculture, as a coping mechanism, exposing themselves to the risk of contracting HIV. The effect was especially observed among non-educated women. A single drought in the last five years increases HIV prevalence in Malawi by around 15% amongst men and women. Overall, the results confirm that weather shocks are important drivers of risky sexual behaviours of young women relying on agriculture in Africa. Further research is needed to investigate the most adequate formal shock-coping strategies to be implemented in order to limit the negative consequences of natural disasters on HIV acquisition and transmission.

## Highlights

• We study the effect of the 2015 Malawi drought on transactional sex, STIs and HIV.
• A moderate drought doubles transactional sex among women relying on agriculture.
• A moderate drought increases by 50% transactional sex among men working outside agriculture.
• The effect of drought on transactional sex is higher among uneducated women.
• Each drought increases HIV prevalence by 15% among men and women.

## 1 Introduction

While the lives of girls and women have dramatically improved over the past quarter century, progress toward gender equality has been limited in poorest countries. Girls and women who live in extreme poverty, in remote areas or who are marginalised continue to lag behind their male counterparts (World Bank, 2016). Globally, women are vulnerable to extreme poverty because they face a greater burden of unpaid work and have limited access to productive assets (Wanjala, 2021). Extreme poverty of women seems to interrelate strongly with infectious diseases, a fact that has motivated several studies to address the link between poverty and risky sexual behaviours through cash transfers targeting adolescent girls (Baird, 2012; Pettifor, 2016). Women are disproportionately affected by sexually transmitted infections (STIs) and HIV/AIDS is currently the leading cause of death worldwide for women aged 15–44 years. Young women aged 15–24 are three times more likely to be infected with HIV than their male counterparts and comprise 31% of all new HIV infections in Sub-Saharan Africa (UNAIDS, 2017). In addition, STIs not only affect women and their sexual partners, they are a special concern during pregnancy and pose important public health threats to unborn children since they are associated with adverse pregnancy outcomes that include spontaneous abortion, stillbirth, prematurity, low birth weight, and multiple sequelae in surviving neonates including HIV and STI (Adachi et al., 2018; Silver et al., 2014; Gomez et al., 2013; de Attayade et al., 2011). Hence reducing STIs and HIV in women will likely translate into large societal benefits that outweigh the costs of such interventions.

While almost 2 million persons become newly infected with HIV each year (UNAIDS, 2017), recent literature has developed an entire conceptual framework in order to better understand the structural drivers of the HIV epidemic among women (Gafos et al., 2020). There are a growing number of studies suggesting that transactional sex – defined as non-marital non-commercial sexual relationships motivated by the implicit assumption that sex will be exchanged for material support or other benefits - is associated with risk of HIV acquisition (Dunkle et al., 2004; Kilburn et al., 2018; Ranganathan et al., 2016). This increased risk can be explained by the partner age difference and the number of different partners of young women engaging in such behaviour (Ranganathan et al., 1999; Collinson et al., 2007). Further, when women are in a situation of monetary dependence with respect to a sexual partner, their bargaining power is reduced, which increases the risk of HIV infection (Ranganathan et al., 2017). Therefore, transactional and commercial sex, alongside with biological susceptibility, are key behavioural practices responsible for gender inequalities in HIV/AIDS (Stoebenau et al., 2016; UNAIDS, 2017). In a recent literature review, Cust et al. (2021) showed that economic shocks are likely to affect both the extensive margin (the number of people engaging in transactional and commercial sex), the intensive margin (the number of sex acts within a relationship) and the degree of riskiness of sexual intercourses. In fact, a number of economic studies have shown that women who engage in transactional and commercial sex may adopt risky sexual behaviours, like unprotected sex, in order to cope with negative income shocks (Burke et al., 2015; De Walque et al., 2014; Dupas & Robinson, 2012; Robinson & Yeh, 2011, 2012). This is explained by the fact that since men have a stronger preference for unprotected sex acts, there is a positive premium for unprotected sex acts (Arunachalam & Shah, 2013; Rao et al., 2003). As for women engaging in transactional sex, Ranganathan et al. (2017) highlighted that when they are relying on material and financial support of their partner, this power imbalance leads to a higher risk of HIV infection. In addition, women experiencing income shocks may be unable to afford treatment for STIs, making them more vulnerable to HIV since the presence of any STI increases both the risk of new infections among HIV-negative people and the risk of transmission from HIV-positive people (Galvin & Cohen, 2004). Economic shocks may also affect immune system directly through increased stress (Segerstrom & Miller, 2004), making them more vulnerable to STIs and HIV.

Income volatility, as opposed to income level and poverty, has only been examined as a structural driver of HIV in recent years and only six published (Burke et al., 2015; Dinkelman et al., 2008; Dupas & Robinson, 2012; Robinson & Yeh, 2011, 2012; Wilson, 2012) and two unpublished studies (De Walque et al., 2014; Tenikue & Tequame, 2018) have investigated the sexual response of women to negative economic shocks, and among these, three focused on behaviours of sex workers (Dupas & Robinson, 2012; Robinson & Yeh, 2011, 2012). Among these, the study of Burke et al. (2015) used data from 19 African countries and showed that rainfall shocks (droughts) increase HIV prevalence by 11%. The magnitude of this effect is meaningful, considering that droughts are common in Africa. Based on their model, authors estimate that drought-induced income shocks lead to a 17% increase in HIV prevalence over a 10-year period. If the size of the effect is taken at face value, it would mean that income volatility is one of the main drivers of HIV in Africa. However, despite the important policy implications of such results, there is still a weak understanding of the channels through which a drought affects HIV. In fact, while it is generally assumed that transactional sex is an important transmission channel, the authors could not test this given that information on transactional sex was only added in DHS survey after 2015. The present article seeks to make an important contribution to the literature. It contributes to a growing body of evidence that extreme weather events can increase HIV prevalence, by analysing the effects of drought on risky behaviours of men and women. Precisely, it explores whether a main transmission channel between drought and HIV is transactional sex, which has not yet been shown in the literature.

## 2 Theoretical framework

Following Burke et al. (2015),^1^ we consider the relation between droughts and transactional sex and finally between droughts and HIV prevalence.

We first consider how this relationship operates for women.

First, droughts can generate economic shocks for individuals whose primary income source is agriculture, by affecting agricultural outputs. Droughts may also generate economic shocks more widely through the channel of increasing food prices, but empirical evidence suggests that the effects of recent droughts in Malawi have been concentrated amongst individuals with agricultural incomes (Pauw et al., 2011; Loevinsohn, 2015; World Bank, 2016). We therefore follow Burke et al. (2015) in hypothesizing that the incomes of women working in agriculture are more sensitive to drought than women working outside agriculture. Second, we hypothesize that the supply curve for transactional sex shifts outwards in response to an economic shock because it allows women to smooth consumption by raising money quickly (Lo Piccalo et al., 2012). Finally, the risk of HIV is increasing due to potential increase in multiple concurrent sexual partners, disassortative sexual mixing and unprotected sex (Downs & De Vincenzi, 1996; Hertog, 2007; Mah & Halperin, 2010, Ranganathan et al., 1999). Women engaging in transactional sex are more likely to have multiple concurrent partners, some or all of whom provide them with material support (Okigbo et al., 2014; Ranganathan et al., 2017). The sexual partners of women engaging in transactional sex may also be more likely to be infected with HIV than average since they are older men, with greater material resources, or the clients of sex workers (see Kilburn et al., 2018). Power relations and the premium on condom-less sex make unprotected sex acts more likely within transactional relationships than regular relationships (MacPherson et al., 2012; Ranganathan et al., 2017).

We next consider how the relationship operates for men, in order to understand behaviours of the demand side of the market. It should be noted that men might also engage in transactional sex, albeit with a lower prevalence than women (Wamoyi et al., 1999). However, the data available in the Malawi DHS 2015-16 focuses only on men's consumption of transactional sex.

Again, we consider the sequential relation going from drought to economic shocks to transactional sex and finally to HIV prevalence. First, for men employed outside agriculture, the effect of drought on income will be smaller than the effect of drought for women employed in agriculture. It may also be that the effect of drought will be smaller for men than for women in the same type of employment, if men are better insured against shocks (Dercon & Krishnan, 2000; Gong et al., 2019). If this is the case, men working in agriculture could also increase their consumption of transactional sex in equilibrium. Second, a decrease in income will cause an inwards shift in the demand curve for transactional sex. The equilibrium outcome for consumption of transactional sex depends on the relative sizes of this inwards shift, and the outwards shift in the supply curve. Indeed, the increase in supply is likely to push prices down which in turn may maintain or raise the demand for transactional sex by men (even if their own income is reduced), this depending on the price elasticity of transactional sex. Finally, although transactional sex is primarily considered a risky behaviour for women, engaging in transactional sex may also increase men's risk of contracting HIV, given that they are more likely to have multiple concurrent partners or are less likely to use condoms in transactional relationships (Chopra et al., 2009; Maganja et al., 2007; Serwadda et al., 1992).

The new equilibrium outcomes expected following a drought-related economic shock are as follows:

H1 Women employed in agriculture increase their supply of transactional sex in areas affected by droughts.

H2 Men employed outside agriculture increase their consumption of transactional sex in areas affected by droughts.

H3 Rates of STI infections are higher amongst men and women in drought-affected areas.

The STI-related predictions on employment type are ambiguous. We expect women employed in agriculture and men employed outside agriculture to be more likely to be infected with a STI as a result of transactional sex, but these individuals will simultaneously expose their other present and future sexual partners who may be employed in both types of occupation.

## 3 Material and methods

### 3.1 Data and descriptive statistics

#### 3.1.1 Study setting

Malawi is a small country in Southern Africa, with a population of approximately 17.6 million people. In 2016, the HIV prevalence rate amongst adults aged 15–49 was 9.2% - slightly higher than the regional average of 7.0% (NSO, 2017). The practice of transactional sex has been well documented in Malawi; women may exchange sex for material support, employment, cash or to pay off debts (Loevinsohn, 2015; MacPherson et al., 2012; Swidler & Watkins, 2007). From 2014 to 2016, Malawi experienced major droughts due to unusually strong El Niño conditions. In 2015, Malawi experienced one of the worst flood in its history that was followed by a drought that started in October 2015 and ended in March 2016, coinciding with the timing of data collection of the DHS. The “state of national disaster” was declared in October 2015 and USD 149.36 millions were mobilized to provide financial assistance to affected districts.

As Table 1 shows, during the rainy seasons of November 2014–April 2015 and November 2015–April 2016, approximately 90% of the population experienced a drought of various intensities.

**Table 1:** Length of moderate droughts during the 2015–2016 rainy season.

Number of months	All women	“Tran” Women	Men
0	8.03	5.04	8.12
1	5.27	3.43	6.04
2	1.53	1.03	1.55
3	0.09	0.00	0.11
4	4.81	4.33	5.85
5	0.81	0.62	0.74
6	79.46	85.55	77.58
Obs.	24,562	1,863	6,393

Notes: Percentages are presented in this table.

Estimates are weighted to be representative at the national level.

### 3.1.2 Individual data

DHS data collection took place between October 2015 and February 2016, in Malawi. DHS surveys administer interviews on a wide range of topics in population and health. In 2015, a question on transactional sex practices was introduced into the interviews in Malawi for the first time among a subsample of unmarried women aged 15 to 24. The coincidence of the timing of the drought and survey allows us to investigate the channel of transmission between drought and HIV, by comparing the transactional sex practices of individuals affected by drought with those who were not affected.

The 2015-16 DHS survey in Malawi collected data from interviews with a sample of 24,562 women and 7,478 men. To allow for construction of nationally representative data, the sample was stratified by the 28 districts of Malawi, and then by urban and rural areas, resulting in 56 strata. Within these, the survey clusters of households were randomly sampled based on population census enumeration areas, and then households within the sampled clusters were randomly selected for interview. All women aged 15–49 in the selected households were eligible for individual interviews. A random sample of one third of these households was also eligible for individual interviews of male residents aged 15–54; and collection of blood samples from men and women for voluntary HIV testing.

All women aged 15–24 who ever had sex, and were not married or living with a partner at the time of the survey, were asked the question *“In the past* *twelve* *months* *have you had sex or been sexually involved with anyone because he gave you or told you he would give you gifts, cash, or anything else”* (NSO, 2017). 1,863 women answered this question. All men aged 15–54 who ever had sex were asked if, in the last twelve months, they had *“given any gifts or other goods in order to have sex or to become sexually involved with anyone”*, and *“paid anyone in exchange for having sexual intercourse”*. The former question may be considered a conservative estimate of transactional sex, and we combined both outcomes in the empirical analysis.

Women and men age 15–49 years who had worked in the past twelve months were asked to declare their main occupation over that period.^2^ We use the answer provided to this question to determine if each respondent work in or outside the agricultural sector.

Data on HIV prevalence was collected by taking voluntary blood samples from all adults in the eligible third of households. In total, HIV testing was carried out on blood samples from 7,718 women and 6,593 men. By employing cluster-specific inverse-probability sampling weights, the HIV prevalence rates estimated are representative at the national level. Testing for HIV was carried out using the DHS anonymous linked protocol, which allows for the merger of HIV test results with the sociodemographic data collected in individual interviews after the removal of all information which could potentially identify an individual (NSO, 2017). Testing was carried out anonymously, and therefore individuals cannot be provided with their results, but all respondents – whether they volunteered to provide blood samples or not - were provided with educational materials and offered referrals for free voluntary counselling and testing.

**Fig. 1:**
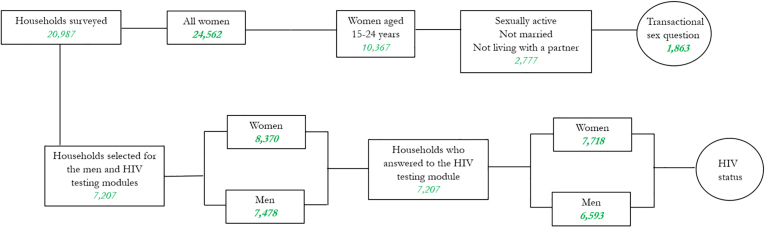
Sample.

Fig. 1 summarizes the samples for which different information is available.

DHS surveys do not collect data on households' income. However, longitude and latitude information about the location of each cluster is recorded, making it possible to match responses to data on local weather conditions. Variation in weather is frequently used in economics literature as a proxy for variation in income at the local level, particularly in SSA where the majority of individuals depend on agriculture for their livelihood (Davis et al., 2010).

#### 3.1.3 Weather data

**Fig. 2:**
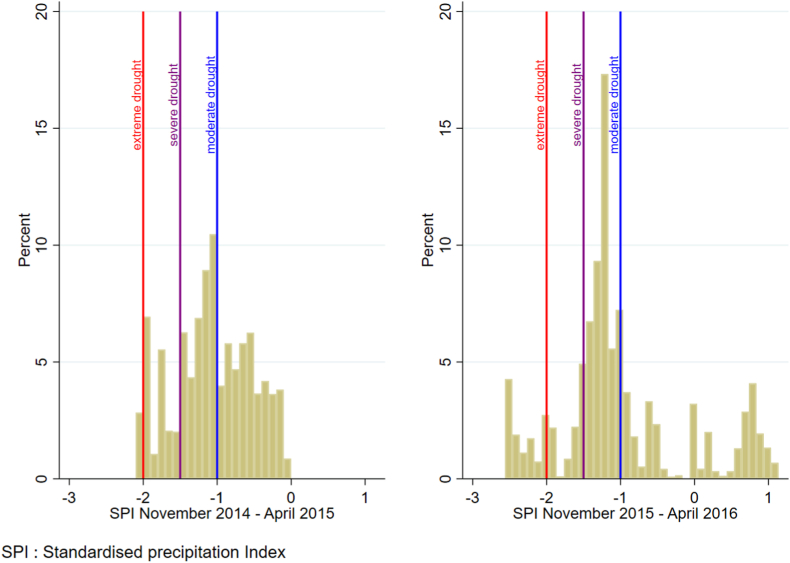
Histograms of Standardised Precipitation Index by cluster (average over the period). Notes: These two histograms are based on the average SPI in that six months period.

We used data from the Global Precipitation Climate Centre monthly precipitation dataset to built the Standardised Precipitation Index (SPI) (Schneider et al., 2011), which is a measure of precipitation that expresses observed precipitation in terms of deviation from the long-term climatological average at a given location. It is therefore, by construction, orthogonal to confounding variables correlated with absolute rainfall levels. The data were available at a 0.5° resolution until 2016 and a 1.0° resolution from 2017 to 2018, and matched to the longitude and latitude of each DHS cluster. The SPI was obtained by fitting a gamma probability density function to long-term precipitation for a chosen time scale, which was then transformed to a normal distribution. The SPI was calculated over a 6-month time scale (November–April) during Malawi's annual rainy season, when main crop-growing activities take place (World Bank, 2016). This allows capturing precipitation of relevance to agricultural output. We use a reference period of almost 60 years when constructing the SPI (a minimum of 30 years is recommended by Guttman (1999)). The weather level data was constructed using the reference period 1951–2018, in order to capture recent climatic changes (Burke et al., 2015). Fig. 2 shows the distribution of the SPI across DHS clusters during the 2014-15 and 2015-16 seasons. Table 1 displays the duration in months of the moderate droughts during the 2015–2016 rainy season based on DHS information.

Following McKee and Doesken (1995), we define a drought when precipitation falls to one standard deviation below the long-term average, and we consider it ends when it returns to a positive value. This enables us to compute the length of the drought in number of months. Every cluster where precipitation fell to this level during the six months period going from November to April is considered to have experienced a drought during the (agricultural) year. Through the properties of the normal distribution, an SPI of minus one is equivalent to an event that occurs with approximately 16% probability during the reference period. As such, our measure of drought is comparable with the definition used by other authors examining the effect of economic shocks on HIV and transactional sex of a crop-year rainfall realisation below the 15% quantile of the local rainfall distribution (Burke et al., 2015; Low et al., 2019). This threshold was selected as meaningful by Burke et al. (2015) because realizations below the 15th percentile appear to be the most harmful to maize yields which is the main staple food crop in Malawi (World Bank, 2016). In our robustness checks, we show that our results are unchanged when considering the effect of severe drought, which is defined as starting at -1.5 standard deviations from the mean (McKee & Doesken, 1995).

By construction, each cluster has an equal chance of experiencing drought in any given year. It is essential for the identification strategy to use a measure of a drought relative to local conditions, as opposed to an absolute measure. Using an absolute measure of drought would mean that clusters with lower or more variable rainfall would expect more shocks; these clusters could differ in other observable and unobservable ways that affect transactional sex and HIV. Using a relative measure also captures plausibly the rainfall realizations that constitute economic shocks. For example, farmers in areas with lower historical rainfall may have adapted to this by growing crops such as sorghum that yield less than maize, but are more resistant to drought.

In the context of climate change, there could be concerns that some parts of Malawi have recently begun to experience lower or more variable rainfall than others and that this trend is not reflected in the long-term mean. If recent climatic changes were correlated with transactional sex or HIV, this could introduce bias into the results. However, by virtue of its small size and limited geographic diversity, Malawi is unlikely to be unevenly affected by climate change.

**Fig. 3:**
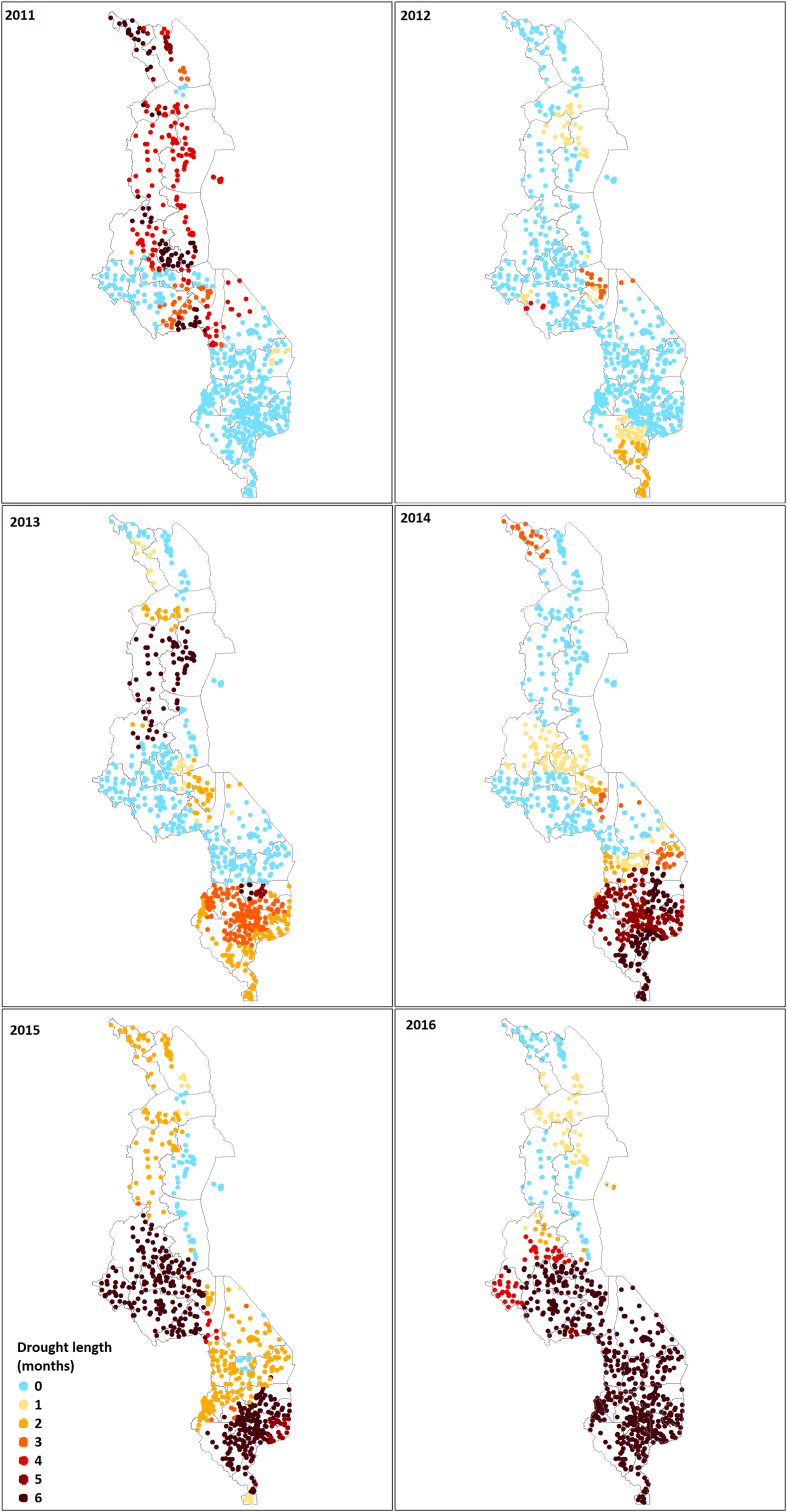
Number of months that DHS interview clusters were affected by a moderate drought between 2011 and 2016.

Fig. 3 shows that all regions of the country experienced a drought in the rainy seasons between 2011 and 2016. In addition, it shows that the duration of the drought also seems to vary over time, since we can see that in 2011 and 2013 the north of the country experienced a long drought while between 2014 and 2016, it was mostly the regions located in the South that experienced a longer drought. In addition, the Appendix, Table A1 shows that there is limited positive correlation between the recent droughts in Malawi, as well as frequent negative correlations. Together, this information builds confidence that drought in Malawi is a random event occurring in any part the country.

### 3.2 Empirical strategy

#### 3.2.1 Effect of drought on transactional sex

In order to understand whether current drought affects current transactional sex practices (and to test hypotheses H1 and H2 from the theoretical framework section), we consider as the main variable of interest the ongoing drought during the survey period (i.e. occurring during the rainy season of November 2015 to April 2016) and which overlapped with the twelve months prior to interview (i.e. occurring during the rainy season of November 2014 to April 2015). We estimate these relationships by regressing the transactional sex variable on current droughts as indicated in the following equation:

(2) $$Y_{ij}=\alpha_{1}+\beta_{1}\;S_{j}^{t}+\delta_{1}a_{i}+\gamma_{1}r_{j}+\omega +\varepsilon_{1ij}$$

where *Y* is a binary outcome equal to 1 if individual *i* in cluster *j* engaged in transactional sex in the past twelve months. $S_{j}^{t}$ is a categorical variable equal to one if cluster *j* experienced a moderate drought lasting between one and five months, equal to 2 if the drought lasted six months and equals to 0 if there was no drought experienced in year *t*. We also explore the timing of the relationships between the outcomes of interest and drought, by running two alternative regressions where $S_{j}^{t}$ is a dummy variable taking value one if there was a six-months drought during the 2015–2016 and 2014–2015 rainy seasons respectively. $a_{i}$ refers to the individual's age and $r_{j}$ indicates whether the individual lives in a rural cluster or not. ω is a vector of interview-month fixed affects, and $\varepsilon_{1ij}$ is a mean-zero error term. We estimate these equations separately for men and women working in and outside agriculture, in order to test the predictions that increases in transactional sex are concentrated in women in agriculture and men outside agriculture.

We estimate linear probability models allowing for correlation of the error term across individuals in the same weather grid (cluster). We use inverse-probability sampling weights to make the results representative at the population level.

#### 3.2.2 Effect of drought on STI and HIV status

In order to confirm whether current drought increases current risk of HIV through the channel of transactional sex, we also explore the effect of these droughts on whether an individual experienced an STI in the last twelve months using the same identification strategy as the one used for transactional sex. Indeed, treatable STIs are a proxy for current exposure to risk of contracting HIV and also increase biological susceptibility to infection (Galvin & Cohen, 2004).

Furthermore, the link between current behaviours and current HIV infection may be difficult to identify for the following reasons: (i) the probability of being infected with STI and HIV is small for each unprotected sex act, (ii) there is a delay between risky behaviours occurring, and manifesting in statistically identifiable increases in HIV infections at the population level^3^ and (iii) a HIV infection could have been acquired at any time in the past.

We therefore followed Burke et al. (2015) to explore the impact of droughts up to ten years in the past on present health status, and thus regress HIV status on the number of six-months droughts in the past two, five and ten years:

(3) $$Y_{ij}=\alpha_{1}+\beta_{2}\;S_{j}^{n}+\delta_{2}a_{i}+\gamma_{2}r_{j}+\omega +\varepsilon_{2ij}$$

where *Y* is a binary outcome equal to 1 if individual *i* in cluster *j* is HIV positive. $S_{j}^{n}$ is a continuous variable indicating the number of six-months droughts the cluster *j* experienced in the last *n* years. As previously we estimate these equations separately for men and women, and then for men and women by occupation type, in order to test the predictions presented in section 2.

Again, we estimate linear probability models allowing for correlation of the error term across individuals in the same cluster. We use inverse-probability sampling weights to make the results representative at the population level.

## 4 Results

### 4.1 Descriptive statistics

Table 2 presents descriptive statistics of our sample on sociodemographic characteristics, drought outcomes, sexual risk and infection and alternative coping mechanisms breaking down by gender. On average, women with information on transactional sex were 19 years old and none of them was married. They live in household of six persons and 15% are the household head. Seventy-seven percent lived in a rural area and 31% were relying on agriculture. Eighty-six percent were living in an area affected by a moderate drought in 2015-16 during a period of six months. Given the large proportion of participants who were affected by a drought, we create a categorical variable capturing the presence and the length of the drought. In this specific sample of women, almost 8% were HIV positive, 2% had an STI over the last year and 5% reported to engage in transactional sex. Table 2 also reports descriptive statistics for i) the sample of all women regardless of whether they answered to the transactional sex question or not and ii) the men sample. For this latter, we can note that almost 11% of men declare that they have given cash, gifts or other goods in exchange of sex in the last twelve months.

**Table 2:** Descriptive statistics.

	All women		bl	“Tran” Women ⋄		bl	Men	
Variables	Obs	Mean		Obs	Mean		Obs	Mean
Sociodemographic characteristics								
Age (in years)	24,562	28.11		1,863	19.12		7,478	28.88
Lives in rural location (%)	24,562	81.70		1,863	76.66		7,478	81.51
Is currently married (%)	24,562	61.68		1,863	0.00		7,478	54.06
Education level (in years)	24,562	5.96		1,863	7.33		7,478	7.06
Household size	24,562	5.36		1,863	5.79		7,478	5.28
Is the HH head (%)	24,562	18.27		1,863	15.35		7,478	58.29
Employed in agricultural activities (%)	24,562	39.44		1,863	30.72		7,478	38.47
HH head is employed in agricultural activities † (%)	21,368	39.16		1,190	34.56		6,373	41.29
Drought outcomes ∓								
Drought in 2015–2016								
Experienced no drought	24,562	8.03		1,863	5.05		7,478	8.36
Experienced 1 to 5 months drought	24,562	12.51		1,863	9.40		7,478	14.64
Experienced 6-months drought	24,562	79.46		1,863	85.55		7,478	77.00
Drought in 2014–2015								
Experienced no drought	24,562	4.34		1,863	4.30		7,478	4.45
Experienced 1 to 5 months drought	24,562	34.21		1,863	34.56		7,478	33.64
Experienced 6-months drought	24,562	61.46		1,863	61.14		7,478	61.91
Number of 6-months droughts:								
in the last 2 years	24,562	1.41		1,863	1.47		7,478	1.39
in the last 5 years	24,562	1.64		1,863	1.70		7,478	1.62
in the last 10 years	24.562	1.80		1,863	1.80		7,478	1.79
Sexual risk and infection outcomes								
Tested positive for HIV (%)	7,718	10.96		591	7.79		6,593	7.24
Had STI in the last 12 months (%)	24,507	2.46		1,854	2.01		7,456	2.00
Engaged in transactional sex (%)	–	–		1,863	5.18		6,393	11.02

Notes: Estimates are weighted to be representative at the national level.

∓ A moderate drought is defined by the SPI reaching more than one standard deviation below the long term mean in the 6 month period of interest.

† We have the information on head of HH employment sector only if the interviewed person is the head or his/her spouse.

We can then input the head of HH occupation for other members of the HH.

⋄ Women who answered to the transactional sex question.

### 4.2 Effect of drought on transactional sex

Results presented in Table 3 show that women relying on agriculture in an area affected by a drought of 1–5 months were more likely to engage in transactional sex by almost 5 percentage points and those affected by a six-months drought were 6 percentage points more likely to engage in a transactional relationship in comparison to women living in area who were not affected by a drought. Given the share of individuals declaring such activity in this survey, suffering from a current six-months drought doubles the likelihood of engaging in transactional sex. No effect is detected for women whose main livelihood is not agriculture, either employed or whose household head is employed in another sector. We can note that similar results are found when considering a severe drought instead of a moderate drought (cf. Panel D). Women working outside agriculture are less likely to engage in transactional sex if exposed to a drought between November 2014 and April 2015 (cf. Panel C). We explore whether this result was caused by a switch in occupation in the places affected by a drought in the past. and we find no evidence of such mechanism (see Table A4 (Panel B) in the Appendices). While our results might be biased in the presence of migration, for instance if women living in drought-stricken areas who have the opportunity to migrate in order to find alternative sources of income following a drought do migrate, we do not find any evidence of such selection problem as the share of women born in their current place of residence and working in the agriculture sector does not decrease with past extreme climate events (cf. Table A4 in the appendices).

**Table 3:** Effect of droughts on women’s transactional sex in the last 12 months.

	Women sample				
		work in		work outside	
		agriculture †		agriculture ‡	
	All	self	HH head	self	HH head
	(1)	(2)	(3)	(4)	(5)
Main results					
Panel A: Moderate drought in 2015–2016 ⋄					
Ref: No drought					
One to five months of drought	−0.010	0.048∗∗	0.006	−0.024	−0.026
	(0.029)	(0.023)	(0.045)	(0.056)	(0.045)
Six-months drought	−0.008	0.060∗∗∗	0.048	−0.006	−0.026
	(0.026)	(0.017)	(0.047)	(0.055)	(0.044)
Observations	1,863	532	389	425	624
R2	0.007	0.023	0.023	0.016	0.019
Average of transactional sex	0.052	0.057	0.064	0.062	0.041
Robustness checks					
Panel B: Moderate drought in 2015–2016					
Six-months drought (=1)	−0.001	0.023	0.044	0.011	−0.010
	(0.016)	(0.023)	(0.027)	(0.033)	(0.026)
Panel C: Moderate drought in 2014–2015					
Six-months drought (=1)	−0.023	−0.005	−0.022	−0.079∗	−0.078∗∗∗
	(0.016)	(0.025)	(0.032)	(0.042)	(0.030)
Panel D: Severe drought in 2015–2016					
Ref: No drought					
One to five months of drought	−0.002	0.089∗∗∗	0.033	−0.054	−0.054
	(0.025)	(0.030)	(0.046)	(0.067)	(0.039)
Six-months drought	−0.006	0.050∗∗∗	0.034	−0.052	−0.042
	(0.022)	(0.015)	(0.034)	(0.070)	(0.041)

Clustered standard errors in parentheses. ∗ p < 0.10, ∗∗ p < 0.05, ∗∗∗ p < 0.01.

⋄ Droughts from November to April are considered.

† work in agriculture as self-employed or employee. ‡ work outside agriculture: professional/technical/managerial work, clerical, sales, household and domestic, services, skilled and unskilled manual work). Individuals who do not work are excluded from the sub-group analysis which explains that the number of observations of (1) is different from the addition of observations in (2) and (4). Occupation of the HH head was not available for all individuals.

As for results presented in Table 4, they show that a drought increases transactional sex relationships for all men but the effect is slightly stronger among men working outside agriculture. Precisely, we find that men living in areas affected by a six-months drought are 5 percentage points more likely to engage in a transactional relationship with a woman than men living in areas unaffected by the drought. It is interesting to note however that there is no effect of shorter droughts (1–5 months) on men's engagement in a transactional sex relationship. Similar results are obtained when considering severe droughts (cf. Panel D in Table 4) or when comparing individuals living in areas where a six-months moderate drought occurred (cf. Panel B in Table 4).

**Table 4:** Effect of droughts on men’s transactional sex in the last 12 months.

	Men sample				
		work in		work outside	
		agriculture †		agriculture ‡	
	All	self	HH head	self	HH head
	(1)	(2)	(3)	(4)	(5)
Main results					
Panel A: Moderate drought in 2015–2016 ⋄					
Ref: No drought					
One to five months of drought	0.002	−0.023	−0.007	0.026	−0.003
	(0.016)	(0.021)	(0.023)	(0.024)	(0.022)
Six months drought	0.056∗∗∗	0.037∗	0.052∗∗	0.064∗∗∗	0.051∗∗
	(0.014)	(0.021)	(0.022)	(0.018)	(0.020)
Observations	6,393	2,585	2,261	3,216	3,074
R2	0.012	0.018	0.013	0.016	0.010
Average of transactional sex	0.110	0.102	0.094	0.122	0.107
Robustness checks					
Panel B: Moderate drought in 2015–2016					
Six-months drought (=1)	0.055∗∗∗	0.054∗∗∗	0.057∗∗∗	0.050∗∗∗	0.053∗∗∗
	(0.011)	(0.014)	(0.015)	(0.015)	(0.014)
Panel C: Moderate drought in 2014–2015					
Six-months drought (=1)	−0.033∗∗∗	−0.074∗∗∗	−0.059∗∗∗	−0.005	0.008
	(0.012)	(0.019)	(0.018)	(0.016)	(0.016)
Panel D: Severe drought in 2015–2016					
Ref: No drought					
One to five months of drought	−0.004	0.020	0.003	−0.033	−0.014
	(0.015)	(0.020)	(0.021)	(0.023)	(0.021)
Six months drought	0.042∗∗∗	0.042∗∗	0.031	0.031	0.040∗
	(0.015)	(0.018)	(0.020)	(0.025)	(0.022)

Clustered standard errors in parentheses. ∗ p < 0.10, ∗∗ p < 0.05, ∗∗∗ p < 0.01.

⋄ Droughts from November to April are considered.

† work in agriculture as self-employed or employee. ‡ work outside agriculture: professional/technical/managerial work, clerical, sales, household and domestic, services, skilled and unskilled manual work). Individuals who do not work are excluded from the sub-group analysis which explains that the number of observations of (1) is different from the addition of observations in (2) and (4). Occupation of the HH head was not available for all individuals.

### 4.3 Exploring characteristics of women engaging in transactional sex

After showing the impact of droughts on transactional sex, we now turn to the exploration of the characteristics of women who decide to engage in transactional sex. To do so we consider two different sub-samples: women living in areas (i) where there has been at least one month of drought and (ii) where there has been at least six months of drought. We run a logistic regression including a set of individual (level of education, agriculture occupation, current employment status) and household (wealth index, number of children below five, household size, electricity access, type of floor) characteristics (Gichane et al., 2020; Lépine & Strobl, 2013) and controlled for correlation of the error term across individuals living in the same cluster.

**Fig. 4:**
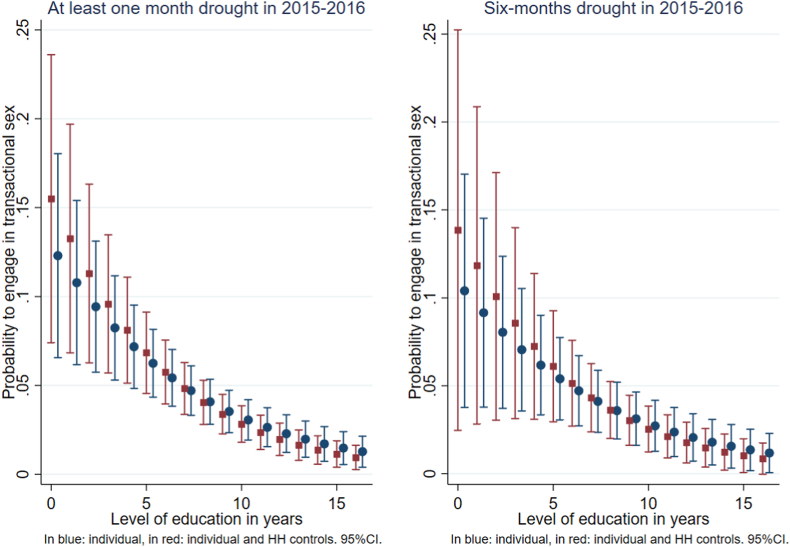
Probability to engage in transactional sex by number of years of education.

From Table 5 we can note that the level of education decreases the likelihood of engaging in transactional sex. This result may reflect different labour opportunities, which is in line with previous evidence (NSO and ICF, 2017), which indicates that women and men with no primary or secondary education most often work in agriculture. Fig. 4 displays the probability of engaging in transactional sex depending on the number of years of education of women. The results were estimated using regressions presented in Table 5.

**Table 5:** Determinants of engaging in transactional sex when suffering from droughts.

	2015–2016			
	1-to-6 months of drought		6-months drought	
Age (in years)	−0.025	−0.004	0.035	0.060
	(0.058)	(0.063)	(0.084)	(0.088)
Education (in years)	−0.150∗∗∗	−0.187∗∗∗	−0.143∗∗∗	−0.185∗∗∗
	(0.034)	(0.039)	(0.045)	(0.059)
Works in agriculture	−0.291	−0.192	0.112	0.288
	(0.394)	(0.394)	(0.513)	(0.417)
Is the HH head	−0.251	0.053	−0.627	−0.154
	(0.390)	(0.475)	(0.659)	(0.742)
Is currently working	0.534	0.469	0.328	0.220
	(0.381)	(0.360)	(0.559)	(0.473)
Rural cluster		−0.357		0.009
		(0.434)		(0.583)
Welath index		0.078		−0.049
		(0.129)		(0.197)
Nb of children below 5		−0.411∗		−0.483∗
		(0.213)		(0.256)
Nb of HH members		0.068		0.133
		(0.088)		(0.141)
HH with access to electricity		0.059		0.233
		(0.551)		(0.715)
Floor made of earth/sand		0.171		−0.318
		(0.393)		(0.572)
Constant	−1.628	−1.950	−2.959∗∗	−3.263∗∗
	(1.063)	(1.192)	(1.485)	(1.576)
Observations	1,611	1,611	902	902
Pseudo R2	0.037	0.052	0.055	0.047
Average of transactional sex	0.048	0.048	0.039	0.039

Clustered standard errors in parentheses. ∗ p < 0.10, ∗∗ p < 0.05, ∗∗∗ p < 0.01.

### 4.4 Effect of drought on STI and HIV

Results presented in Table 6 indicate that currently experiencing a drought significantly increases the likelihood of suffering from a STI symptom in the last twelve months, especially for women who work in the agriculture sector (Panel B). More precisely, the likelihood of suffering from STIs increases by 10 and 12 percentage points (i.e. 43% and 44% increase) among women who work and whose household's head work in the agriculture sector respectively. Such impact of current droughts on STIs is observed among men whose household head works outside the agriculture sector (cf. Table 7).

**Table 6:** Effect of droughts on STIs in the last 12 months - Women sample.

	Women sample				
		work in		work outside	
		agriculture †		agriculture ‡	
	All	self	HH head	self	HH head
	(1)	(2)	(3)	(4)	(5)
Main results					
Panel A: Moderate drought in 2015–2016 ⋄					
Ref: No drought					
One to five months of drought	−0.002	−0.002	0.003	0.000	−0.002
	(0.004)	(0.006)	(0.007)	(0.011)	(0.007)
Six months drought	0.004	0.009	0.013∗∗	−0.002	−0.000
	(0.004)	(0.006)	(0.006)	(0.010)	(0.006)
Observations	24,507	9,350	8,231	6,755	10,613
R2	0.002	0.003	0.002	0.002	0.002
Average STI prevalence	0.025	0.023	0.027	0.031	0.026
Robustness checks					
Panel B: Moderate drought in 2015–2016					
Six-months drought (=1)	0.005∗∗	0.010∗∗∗	0.012∗∗∗	−0.002	0.001
	(0.003)	(0.004)	(0.004)	(0.006)	(0.004)
Panel C: Moderate drought in 2014–2015					
Six-months drought (=1)	0.003	0.004	0.008∗	−0.003	0.001
	(0.002)	(0.004)	(0.004)	(0.005)	(0.004)
Panel D: Severe drought in 2015–2016					
Ref: No drought					
One to five months of drought	−0.002	−0.003	0.001	−0.004	−0.004
	(0.003)	(0.006)	(0.006)	(0.008)	(0.005)
Six months drought	−0.002	0.002	0.010∗	−0.007	−0.007
	(0.003)	(0.005)	(0.006)	(0.008)	(0.005)

Clustered standard errors in parentheses. ∗ p < 0.10, ∗∗ p < 0.05, ∗∗∗ p < 0.01.

⋄ Droughts from November to April are considered.

† work in agriculture as self-employed or employee. ‡ work outside agriculture: professional/technical/managerial work, clerical, sales, household and domestic, services, skilled and unskilled manual work). Individuals who do not work are excluded from the sub-group analysis which explains that the number of observations of (1) is different from the addition of observations in (2) and (4). Occupation of the HH head was not available for all individuals.

**Table 7:** Effect of droughts on STIs in the last 12 months - Men sample.

	Men sample				
		work in		work outside	
		agriculture †		agriculture ‡	
	All	self	HH head	self	HH head
	(1)	(2)	(3)	(4)	(5)
Main results					
Panel A: Moderate drought in 2015–2016 ⋄					
Ref: No drought					
One to five months of drought	−0.002	−0.023	−0.010	0.011	0.013
	(0.007)	(0.014)	(0.012)	(0.011)	(0.010)
Six months drought	0.003	−0.018	−0.004	0.010	0.014∗
	(0.006)	(0.015)	(0.012)	(0.009)	(0.008)
Observations	7,456	2,941	2,533	3,432	3,461
R2	0.003	0.004	0.002	0.005	0.004
Average STI prevalence	0.020	0.014	0.016	0.028	0.025
Robustness checks					
Panel B: Moderate drought in 2015–2016					
Six-months drought (=1)	0.004	−0.001	0.004	0.004	0.006
	(0.004)	(0.006)	(0.006)	(0.007)	(0.006)
Panel C: Moderate drought in 2014–2015					
Six-months drought (=1)	0.002	−0.007	−0.011∗	0.007	0.013∗
	(0.004)	(0.006)	(0.006)	(0.007)	(0.007)
Panel D: Severe drought in 2015–2016					
Ref: No drought					
One to five months of drought	−0.000	−0.002	−0.001	−0.002	0.002
	(0.005)	(0.007)	(0.008)	(0.010)	(0.009)
Six months drought	0.006	0.001	0.001	0.007	0.010
	(0.005)	(0.007)	(0.007)	(0.008)	(0.008)

Clustered standard errors in parentheses. ∗ p < 0.10, ∗∗ p < 0.05, ∗∗∗ p < 0.01.

⋄ Droughts from November to April are considered.

† work in agriculture as self-employed or employee. ‡ work outside agriculture: professional/technical/managerial work, clerical, sales, household and domestic, services, skilled and unskilled manual work). Individuals who do not work are excluded from the sub-group analysis which explains that the number of observations of (1) is different from the addition of observations in (2) and (4). Occupation of the HH head was not available for all individuals.

Table 8 presents the effects of past droughts on HIV prevalence to take into account the cumulative risk of contracting HIV following repeated risky sexual behaviours. To do so we estimate the effect of drought on HIV in the last two, five and ten years. It is interesting to note that an additional six-months drought in the past five years increases the HIV prevalence rate by 1.5 and 1.3 percentage points among women and men respectively. Such figures correspond to an increase of 14% and 18% of HIV prevalence respectively. This increase in HIV prevalence seems to be concentrated among individuals working outside the agricultural sector.

**Table 8:** Effect of past droughts on HIV prevalence.

	Women sample				
		work in		work outside	
		agriculture †		agriculture ‡	
	All	self	HH head	self	HH head
	(1)	(2)	(3)	(4)	(5)
Nb of six-months droughts in the:					
last two years	0.012∗	0.004	0.010	0.006	0.008
	(0.006)	(0.008)	(0.008)	(0.012)	(0.009)
last five years	0.015∗∗∗	0.010	0.015∗	0.019	0.018∗∗
	(0.006)	(0.008)	(0.008)	(0.012)	(0.009)
last ten years	0.003	0.003	0.006	0.006	0.005
	(0.006)	(0.008)	(0.008)	(0.012)	(0.009)
Observations	7,718	2,979	2,693	2,130	3,321
R2	0.053	0.044	0.028	0.063	0.059
Average HIV prevalence	0.110	0.081	0.075	0.160	0.137
	Men sample				
		work in		work outside	
		agriculture †		agriculture ‡	
	All	self	HH head	self	HH head
	(1)	(2)	(3)	(4)	(5)
Nb of six-months droughts in the:					
last two years	0.007	−0.002	0.000	0.015∗	0.012
	(0.005)	(0.008)	(0.008)	(0.009)	(0.009)
last five years	0.013∗∗	0.011	0.009	0.019∗∗	0.017∗∗
	(0.005)	(0.009)	(0.008)	(0.008)	(0.008)
last ten years	0.003	0.002	0.001	0.008	0.005
	(0.005)	(0.008)	(0.007)	(0.008)	(0.008)
Observations	6,593	2,677	2,294	2,950	2,993
R2	0.070	0.042	0.038	0.084	0.085
Average HIV prevalence	0.072	0.053	0.052	0.101	0.099

Clustered standard errors in parentheses. ∗ p < 0.10, ∗∗ p < 0.05, ∗∗∗ p < 0.01.

⋄ Droughts from November to April are considered.

† work in agriculture as self-employed or employee. ‡ work outside agriculture: professional/technical/managerial work, clerical, sales, household and domestic, services, skilled and unskilled manual work). Individuals who do not work are excluded from the sub-group analysis which explains that the number of observations of (1) is different from the addition of observations in (2) and (4). Occupation of the HH head was not available for all individuals.

## 5 Discussion

The results presented in this paper confirm the role of transactional sex as an important transmission channel from a drought to HIV. We find that a drought doubles the likelihood of engaging in transactional sex amongst unmarried young women who work in agriculture but has no effect on sexual behaviour of women working outside agriculture. We were able to identify that this increase in the supply encounter an increased demand for transactional sex that came from both men who worked inside and outside the agricultural sector, although the increase was larger for men outside agriculture, probably because they were less economically affected by the drought than men relying on agriculture. In addition, we showed that the increase in risky sexual practices translated into a greater chance of reporting STIs symptoms in the past twelve months especially for women in agriculture and men outside agriculture.

Our results have an important policy implication. Results highlight the additional vulnerability faced by women relying on agriculture, in particular women who have low education and limited labour opportunities and confirm the results of Chishimba and Wilson (2021) who stress the importance of access to education, assets and non-agricultural opportunities to be able to mitigate the extreme weather events and smooth their consumption. Not only these women have harder living conditions and worse access to health care that are both important drivers of HIV (Gafos et al., 2020), but they are also more affected by natural disasters. The results from this paper suggests that insuring women relying on agriculture against crop failure could be an effective way to prevent them from engaging in risky sexual practices.

This study suffers however from a number of limitations. Firstly, transactional sex was self-reported by male and female participants. It is thus possible that both groups under-declared it, given the sensitive nature of this behaviour. Secondly, while the transactional sex question has been introduced for the first time in the 2015–2016 Malawi DHS, enabling us to study whether natural disasters may increase risk sexual behaviours, this question was not asked to all individuals interviewed but only to unmarried young women sexually active. We acknowledge that such selection in the respondents may lead on the one hand to conservative estimates as men may also engage in transactional sex as suppliers (Quaife et al., 2022) and on the other hand to an overestimation of the effect of drought on transactional sex given the characteristics of the women selected for the transactional sex section (Gichane et al., 2020). In addition, this design reduces substantially the size of our sample and prevent us from investigating whether married or older women also engage in such practice. Married women were asked instead about multiple partnerships. Only around 1% of married women acknowledged having several partners pointing to the social desirability bias inherent in this type of behaviour. Furthermore, the data available prevent us from disentangling the « quantity » (number of acts) and « quality » (unprotected sex) of transactional sex, distinction which is likely to have different impacts on the HIV epidemics. Thirdly, while Austin et al. (2021) highlighted the mechanism that goes from droughts to HIV burden through the food insecurity faced by women, it would have been interesting to investigate the heterogeneity of risky behaviours adopted by women depending on the type of crops they grow (cash crops versus self-consumed crops). Unfortunately, the DHS data at hand does not allow us to explore this dimension. Finally, the data available prevent us from investigating alternative or occasional activities individuals may have in particular during the lean season.

Understanding the potential of shock-coping strategies to help women avoid engaging in risky sexual practices during droughts is a global priority. Over the last years, sub-saharan Africa is experiencing droughts and other weather shocks with increasing frequency (Niang et al., 2014) and 74% of households in Africa report experiencing a weather shock in the last five years (Niles & Salerno, 2018). In addition to weather shocks, at least 60% of African households report large and sudden losses in income every year (Nikoloski et al., 2018), and most of the poorest are not protected by social safety programmes (World Bank, 2016).

The Malawi government implemented cash transfers programs in reaction to the adverse climatic events faced by its population in 2014–2015 and in 2015–2016 and is thinking in implementing a weather index insurance in order to help individuals to face the growing number of climate adverse events (Makaudze, 2018). DHS data indicate that unfortunately this policy had almost no impact as it hardly reached 3% of the population (only 27 out of 807 interviewed households in our sample received the cash transfer in 2014–2015). While different shock coping strategies (cash transfers, food transfers and insurance schemes) could be mobilized by governments, adverse weather events such as droughts impact communities reducing the ability of insurers to offer insurance against these risks and limits the ability of Governments to protect people from these covariant shocks due to the high associated costs required to provide cash transfers to affected communities (in the 2015–2016 drought that hit Malawi 90% of individuals suffered from such natural disaster).

In such context and given the concomitance of shocks (Lazzaroni & Wagner, 2016), individual precautionary saving (Jones & Gong, 2021) or health insurance coverage have more potential to protect people from shocks that can be insured and that are frequent, hence reducing the negative impact of other concomitant covariant economics shocks. Additional evidence is urgently required regarding their effectiveness to prevent HIV.

## 6 Conclusion

We used DHS data collected during the 2015–2016 harsh drought that affected several areas of Malawi to study the effect of an unanticipated economic shock on sexual behaviours of young women and men. We find that amongst women employed in agriculture, the drought doubles the likelihood of engaging in transactional sex. Furthermore, each drought increases HIV prevalence by 15%. While transactional sex is concentrated among women working in the agriculture sector, the consequences of unprotected sex the also spread to women not working in this sector, certainly via the behaviour of the men in their households. Overall, the results suggest that economic shocks are important drivers of risky sexual behaviours among women in Africa. Further research is needed to investigate the most adequate formal shock-coping strategies to be implemented in order to limit the negative consequences of natural disasters on HIV acquisition and transmission.

## Author statement

Carole Treibich: Methodology, Formal analysis, Writing- Reviewing and Editing.

Eleonor Bell: Formal analysis, Writing - Original Draft.

Elodie Blanc: Methodology, Visualization.

Aurélia Lépine: Conceptualization, Supervision, Writing- Reviewing and Editing.

## Financial disclosure statement

None.

## Declaration of competing interest

None.

## Figures and Tables

**Table A1:** Pairwise correlations between droughts over the years

	Number of months of moderate drought over the period November T − 1 - April T									
T	2016	2015	2014	2013	2012	2011	2010	2009	2008	2007
2016	1.000									
2015	0.560∗∗∗	1.000								
2014	0.505∗∗∗	0.291∗∗∗	1.000							
2013	−0.168∗∗∗	−0.023	0.310∗∗∗	1.000						
2012	0.097∗∗∗	0.102∗∗∗	0.141∗∗∗	0.156∗∗∗	1.000					
2011	−0.662∗∗∗	−0.246∗∗∗	−0.566∗∗∗	−0.021	−0.114∗∗∗	1.000				
2010	0.337∗∗∗	0.278∗∗∗	0.276∗∗∗	−0.098∗∗∗	0.317∗∗∗	−0.230∗∗∗	1.000			
2009	−0.322∗∗∗	−0.026	0.035	0.602∗∗∗	0.041	0.155∗∗∗	−0.095∗∗∗	1.000		
2008	0.119∗∗∗	0.238∗∗∗	−0.228∗∗∗	−0.209∗∗∗	0.073∗∗∗	−0.059∗∗	0.043	0.001	1.000	
2007	−0.114∗∗∗	0.007	0.227∗∗∗	0.347∗∗∗	0.282∗∗∗	−0.055∗∗	0.251∗∗∗	0.488∗∗∗	−0.134∗∗∗	1.000
Experienced a 6-months moderate drought over the period November T − 1 - April T										
T	2016	2015	2014	2013	2012	2011	2010	2009	2008	2007
2016	1.000									
2015	0.281∗∗∗	1.000								
2014	0.268∗∗∗	0.296∗∗∗	1.000							
2013	−0.436∗∗∗	−0.231∗∗∗	−0.129∗∗∗	1.000						
2012	–	–	–	–	1.000					
2011	−0.235∗∗∗	0.099∗∗∗	−0.135∗∗∗	−0.038	–	1.000				
2010	0.042	−0.063∗∗	−0.025	−0.020	–	−0.021	1.000			
2009	−0.429∗∗∗	−0.186∗∗∗	−0.115∗∗∗	0.566∗∗∗	–	0.070∗∗	−0.018	1.000		
2008	–	–	–	–	–	–	–	–	1.000	
2007	–	–	–	–	–	–	–	–	–	1.000

Notes: 1,275 clusters. No cluster experienced a 6-months drought during the years 2012, 2008 or 2007.

∗ p < 0.10, ∗∗ p < 0.05, ∗∗∗ p < 0.01.

**Table A2:** Effect of droughts on women’s transactional sex in the last 12 months - robustness checks

	Women sample				
		work in		work outside	
		agriculture †		agriculture ‡	
	All	self	HH head	self	HH head
	(1)	(2)	(3)	(4)	(5)
Main results					
Panel A: Moderate drought in 2015–2016 ⋄					
Ref: No drought					
One to five months of drought	−0.010	0.048∗∗	0.006	−0.024	−0.026
	(0.029)	(0.023)	(0.045)	(0.056)	(0.045)
Six-months drought	−0.008	0.060∗∗∗	0.048	−0.006	−0.026
	(0.026)	(0.017)	(0.047)	(0.055)	(0.044)
Observations	1,863	532	389	425	624
R2	0.007	0.023	0.023	0.016	0.019
Average of transactional sex	0.052	0.057	0.064	0.062	0.041
Panel Abis: Moderate drought in 2015–2016 ⋄					
Ref: No drought					
One to four months of drought	−0.008	0.051∗∗	0.007	−0.021	−0.022
	(0.029)	(0.025)	(0.046)	(0.057)	(0.046)
Five to six months of drought	−0.008	0.059∗∗∗	0.047	−0.006	−0.026
	(0.026)	(0.017)	(0.046)	(0.055)	(0.044)
Panel Ater: Moderate drought in 2015–2016 ⋄					
Ref: No drought					
One to two months of drought	0.019	0.096∗∗	0.086	0.004	−0.014
	(0.032)	(0.048)	(0.075)	(0.061)	(0.052)
Three to six months of drought	−0.010	0.056∗∗∗	0.039	−0.010	−0.027
	(0.026)	(0.016)	(0.046)	(0.055)	(0.043)

Clustered standard errors in parentheses. ∗ p < 0.10, ∗∗ p < 0.05, ∗∗∗ p < 0.01.

⋄ Droughts from November to April are considered.

† work in agriculture as self-employed or employee. ‡ work outside agriculture: professional/technical/managerial work, clerical, sales, household and domestic, services, skilled and unskilled manual work). Individuals who do not work are excluded from the sub-group analysis which explains that the number of observations of (1) is different from the addition of observations in (2) and (4). Occupation of the HH head was not available for all individuals.

**Table A3:** Effect of droughts on men’s transactional sex in the last 12 months - robustness checks

	Men sample				
		work in		work outside	
		agriculture †		agriculture ‡	
	All	self	HH head	self	HH head
	(1)	(2)	(3)	(4)	(5)
Main results					
Panel A: Moderate drought in 2015–2016 ⋄					
Ref: No drought					
One to five months of drought	0.002	−0.023	−0.007	0.026	−0.003
	(0.016)	(0.021)	(0.023)	(0.024)	(0.022)
Six months drought	0.056∗∗∗	0.037∗	0.052∗∗	0.064∗∗∗	0.051∗∗
	(0.014)	(0.021)	(0.022)	(0.018)	(0.020)
Observations	6,393	2,585	2,261	3,216	3,074
R2	0.012	0.018	0.013	0.016	0.010
Average of transactional sex	0.110	0.102	0.094	0.122	0.107
Panel Abis: Moderate drought in 2015–2016 ⋄					
Ref: No drought					
One to four months of drought 1	0.002	−0.023	−0.006	0.025	0.000
	(0.016)	(0.022)	(0.023)	(0.024)	(0.023)
Five to six months of drought	0.055∗∗∗	0.035∗	0.050∗∗	0.064∗∗∗	0.050∗∗
	(0.014)	(0.021)	(0.022)	(0.018)	(0.020)
Panel Ater: Moderate drought in 2015–2016 ⋄					
Ref: No drought					
One to two months of drought	0.007	−0.017	0.007	0.023	0.001
	(0.019)	(0.027)	(0.029)	(0.027)	(0.025)
Three to six months of drought	0.051∗∗∗	0.028	0.042∗∗	0.062∗∗∗	0.048∗∗
	(0.014)	(0.020)	(0.021)	(0.018)	(0.020)

Clustered standard errors in parentheses. ∗ p < 0.10, ∗∗ p < 0.05, ∗∗∗ p < 0.01.

⋄ Droughts from November to April are considered.

† work in agriculture as self-employed or employee. ‡ work outside agriculture: professional/technical/managerial work, clerical, sales, household and domestic, services, skilled and unskilled manual work). Individuals who do not work are excluded from the sub-group analysis which explains that the number of observations of (1) is different from the addition of observations in (2) and (4). Occupation of the HH head was not available for all individuals.

**Table A4:** Place of residence, employment sector and past droughts

		Number of	Number of
	6-months drought	6-months drought	6-months drought
Women sample - By cluster	in 2014–2015	in past 2 years	in past 5 years
Panel A			
Share of individuals born in place of residence	0.050***	0.068***	0.048***
and working in agriculture	(0.017)	(0.011)	(0.010)
Share of individuals born in place of residence	0.030***	0.044***	0.026***
and whose HH head works in agriculture	(0.015)	(0.010)	(0.009)
Panel B			
Share of women	0.011	0.010	0.004
working outside agriculture	(0.014)	(0.019)	(0.009)
Share of women whose head of HH	0.007	0.000	0.008
works outside agriculture	(0.012)	(0.008)	(0.011)

Notes: Coefficients of independent regressions at the cluster level are reported in this Table.

Standard errors in parentheses. *p < 0.10, **p < 0.05, ***p < 0.01.

## Data Availability

Data will be made available on request.

## Appendices

### Formalised theoretical framework

Following Burke et al. (2015), we consider the following relationship between drought-related economic shocks, transactional sex, and HIV prevalence:

(A1) $$\frac{\delta HIV}{\delta S}=\frac{\delta HIV}{\delta p}\;\frac{\delta p}{\delta z}\;\frac{\delta z}{\delta S}$$

where *HIV* is local HIV prevalence, *S* is a measure of drought relative to local conditions, *p* is a measure of sexual risk and *z* is a measure of income.

We first consider how this relationship operates for women:

$\frac{\delta HIV}{\delta p}$ represents how HIV infection rates change in response to sexual risk. The risk of HIV is increasing in factors including multiple concurrent sexual partners, disassortative sexual mixing and unprotected sex (Downs & De Vincenzi, 1996; Hertog, 2007; Mah & Halperin, 2010). Women engaging in transactional sex are more likely to have multiple concurrent partners, some or all of whom provide them with material support (Okigbo et al., 2014). The sexual partners of women engaging in transactional sex may also be more likely to be infected with HIV than average (older men, with greater material resources, or the clients of sex workers) (Kilburn et al., 2018). Power relations and the premium on condom-less sex make unprotected sex acts more likely within transactional relationships (MacPherson et al., 2012).

$\frac{\delta p}{\delta z}$ represents how sexual risk changes in response to changes in income. We hypothesize that the supply curve for transactional sex shifts outwards in response to an economic shock because it allows women to smooth consumption by raising money quickly (LoPiccalo et al., 2012).

$\frac{\delta z}{\delta S}$ represents how income changes in response to drought. Droughts can generate economic shocks for individuals whose primary income source is agriculture, by affecting production. Droughts may also generate economic shocks more widely through the channel of increasing food prices, but empirical evidence suggests that the effects of recent droughts in Malawi have been concentrated amongst individuals with agricultural incomes (World Bank, 2016; Loevinsohn, 2015; Pauw et al., 2011). We therefore follow Burke et al. (2015) in hypothesizing that the incomes of women working in agriculture are more sensitive to drought than women working outside agriculture.

We next consider how the relationship operates for men, in order to understand the demand side of the market. It should be noted that men might also engage in transactional sex on the supply side, albeit with a lower prevalence than women (Wamoyi et al., 2019). However, the data available in the Malawi DHS 2015-16 focuses only on men's consumption of transactional sex.

$\frac{\delta HIV}{\delta p}$: Although transactional sex is primarily considered a risky behaviour for women, engaging in transactional sex may also increase men's risk of contracting HIV, particularly if they have multiple concurrent partners or are less likely to use condoms in transactional relationships (Chopra et al., 2009; Maganja et al., 2007; Serwadda et al., 1992).

$\frac{\delta p}{\delta z}$: A decrease in income will cause an inwards shift in the demand curve for transactional sex. The equilibrium outcome for consumption of transactional sex depends on the relative sizes of this inwards shift, and the outwards shift in the supply curve. Indeed, the increase in supply is likely to push prices down which in turn may maintain or raise the demand for transactional sex by men (even if their own income is reduced), this depending on the price elasticity of transaction sex.

$\frac{\delta z}{\delta S}$: For men employed outside agriculture, the effect of drought on income will be smaller than the effect of drought for women employed in agriculture. It may also be that the effect of drought will be smaller for men than for women in the same type of employment, if men are better insured against shocks (Dercon & Krishnan, 2000). If this is the case, men working in agriculture could also increase their consumption of transactional sex in equilibrium.

## Footnotes

1 A formalised version of the theoretical framework based on the equations presented in Burke et al. (2015) can be found in the appendices.

2 The following occupations are proposed to the interviewed individuals: professional/technical/managerial, clerical, sales and services, skilled manual, unskilled manual, domestic service, agriculture and other.

3 In a model produced by Burke et al. (2015), effects on HIV are first visible two years after drought.
